# Legalism and tobacco control in the EU

**DOI:** 10.1093/eurpub/cky154

**Published:** 2018-11-01

**Authors:** Holly Jarman

**Affiliations:** University of Michigan School of Public Health, Ann Arbor, MI, USA

## Abstract

Tobacco control policies rely heavily on law and are steeped in litigation. Examining the creation of tobacco control laws in the European Union, this article lays out the potential costs and benefits of legalism for public health practitioners and advocates. Legalism is a form of governance that relies heavily on the authority of law to settle political disputes, with consequences for the ways in which governments make, and advocates push for, public health policies. To the extent that legalism creates procedural problems and distortions in the content of public health policy, then the content of law and its making is a public health problem. On the other hand, litigation can also be a public health tool, and an enforceable, justiciable right to health is a powerful political and legal weapon in its own right.

## Introduction

Public health often works through law, regulating industries and individuals and punishing specific actions. It is impossible, therefore, to understand contemporary public health without understanding legalism and its politics.

Legalism is a mode of governance characterized by a strong emphasis on the formulation and enforcement of legal rules over less formal policies, frequent court cases, legal expertise and a multiplicity of formal policymaking structures (such as public consultation or impact assessment) that can help policymakers to manage legal risks.

Officials, researchers and advocates working to address public health problems can benefit from understanding the causes and consequences of legalism, which has become a well-established way of working in several health-related policy areas and looks likely to become the new norm in others. Tobacco control measures rank among the most successful policies in public health. But they are also subject to some of the most intense legal scrutiny and industry use of legal challenges seen by any public health interventions. Examining tobacco control regulations is therefore an excellent way to examine the development of legalism in public health policymaking and its consequences.

Legalism is frequently thought of as a purely American phenomenon. When we think about lengthy, expensive court cases involving tobacco companies, smokers and governments, we are likely to think about the USA. Indeed, until the 1990s, little tobacco-related litigation took place in countries other than the USA. From the 1990s onwards, however, the number of tobacco-related court cases outside of the USA has increased dramatically. In line with this trend, as European Union (EU) institutions began to craft collective tobacco control laws, the number of legal challenges seen in Europe began to increase. In recent years, we have seen a number of highly significant tobacco-related cases involving EU member states. This paper reviews these cases and the surrounding politics in order to shed light on the development and consequences of legalism.

## What is legalism?

Legalism is a form of governance that occurs when the law is viewed as a highly important, or even dominant, form of authority. In a legalist paradigm, societies rely heavily on law and judges to address political conflicts. Politicians, bureaucrats, advocates, business interests and other stakeholders view laws and regulations, courts and the formal bureaucratic procedures that accompany them as essential (and sometimes preferable) parts of the political environment. All actors anticipate legal challenges and act accordingly: politicians prioritize legislation and rulemaking over informal negotiations, bureaucrats oversee the formulation of rules and attempt to manage legal risks, judges interpret policy and policymaking and interest groups formulate and use legal arguments. ‘Soft law’ (standards that govern behavior but which are not, taken alone, legally enforceable) exists, but remains in the shadow of hard law.

Legalism can arise as a result of competing demands in highly contentious political spaces, particularly where other forms of interest aggregation (such as political parties, civil society groups or other mechanisms linking governments, industry representatives and trade unions) are weak or absent. In those circumstances, actors may begin to demand better protection under the law, or seek out legal redress more readily.

Researchers observing legal systems in different countries note that states have different varieties of legalism based on their domestic institutions and dominant values.^1^^,^^2^ The USA, e.g. is home to *adversarial legalism*, characterized by frequent and costly court cases, highly complex regulations that emphasize enforcement, judges that exist outside of state hierarchical controls, and highly mobilized legal actors. Some continental European countries, in contrast, have a long tradition of *bureaucratic legalism*, relying on interpretation of bodies of rules by state-appointed bureaucrats to solve political disputes.^2^ Other EU countries, the UK and Ireland, e.g. have historically shunned legalism in favor of informal policymaking through behind-the-scenes negotiations -in the case of tobacco control, literal ‘smoke-filled rooms’.^3^

EU member states may have different legal styles, but political scientists argue that global forces acting upon them are causing a degree of convergence. In particular, deregulation, followed by the proliferation of legal rules designed to promote and manage cross-border markets, creates ideal conditions for legalism.^4^^,^^5^ As states cannot operate alone in cross-national policy spaces, consolidating markets across borders requires an alternative central authority, a way to set market rules and solve disputes between actors. That authority is often the law, as embodied in organizations such as the World Trade Organization or World Intellectual Property Organization, or the dispute settlement mechanisms contained within international investment agreements.

The EU institutions are a case in point, having developed their own variety of legalism, *Eurolegalism.*^5^ Eurolegalism is characterized by ‘detailed rules containing strict transparency and disclosure requirements; legalistic and adversarial approaches to regulatory enforcement and dispute resolution; slow, costly legal contestation; active judicial review of administrative action; and empowerment of private actors to enforce legal norms’.^6^

Beginning in the 1980s, European states embarked on an ambitious project to create a single market. In doing so, they granted authority to the European institutions to address regulatory barriers to the smooth operation of a fully-integrated market for goods, services and capital. The EU institutions began to pass more, and more detailed, legal rules to govern areas such as environmental policy and public health that could potentially be barriers to the single market. The EU’s supranational court, the European Court of Justice (ECJ) (which later became the center of an EU court system) played an active role in settling disputes about EU-wide regulations. Over time, the EU’s legal institutions have responded to a whole host of competing demands, not just for or against economic aspects of market integration, but concerning the fundamentals of a variety of social policies and citizenship rights.^7^

## How has legalism played out in EU tobacco control policy?

In EU tobacco control policy, competing demands for market integration and calls for stronger public health protections have been formalized in law, resulting in a policy environment strongly shaped by market regulations and the risks and opportunities inherent in legal actions.

The Single European Act (SEA) signed in 1986 was a fundamental treaty that laid out the path to the creation of a European single market. The SEA authorized the European Commission to take actions to facilitate market integration and recognized that the Commission could address regulations designed to protect health in that context.^8^ But the treaty did not authorize the Commission to pursue public health protections outside of its market integration activities. Subsequent treaties have incrementally strengthened the legal basis for health actions in the EU, but the market-centric tenor of the SEA and subsequent treaties fundamentally shaped tobacco control policymaking for good.

The Commission acted quickly to capitalize on its new authority. The first EC-wide laws addressing tobacco control, designed to harmonize policies among member states, banned television advertizing of tobacco products (89/552/EEC), addressed health warnings on tobacco packaging (89/622/EEC), set maximum levels of tar in cigarettes (90/239/EEC), extended the labeling directive (leading to a ban on snus in all member states except Sweden, 92/41/EEC) and required minimum taxation levels for tobacco products (92/12/EEC, 92/79/EEC, 95/59/EEC).^9^

Although the implementation of the labeling directive in Italy was challenged in court (C-222/91), it was a further, more ambitious Directive that caused the most trouble for the Commission. Directive 98/43/EC banned all tobacco advertizing across the EU. It took almost 10 years to pass, in some part thanks to the lack of a clear legal base in the treaties, and upon passage was immediately challenged by Germany in the ECJ on the grounds that the rules went further than necessary to promote the smooth operation of the single market. In a blow to the aspirations of tobacco control advocates, the Court annulled the advertizing directive in 2000 but also went further to suggest how the problem could be addressed.^10^^,^^11^

Several factors contributed to the fragility of EU tobacco control policy in this early period. The EU Commission, often characterized as the ‘policy engine’ of the EU, did not have a dedicated health directorate until DG SANCO was established in 1999. Public health interest groups were also a comparatively weak presence in Brussels, and so decisions on the first round of directives were made in relative isolation by EU officials.^9^ Over time, however, public health groups mobilized at the EU level and health became a broader part of the Commission’s agenda.

Crucially, a number of key member states that initially opposed the formulation of a comprehensive tobacco control law at the EU level (realized as the Tobacco Products Directive (TPD), 2001/37/EC) weakened their opposition.^12^ Economically, this change of heart was driven by states’ decreasing reliance on the tobacco industry as a source of employment and revenue. Politically, the election of left-of-center parties that were more supportive of anti-tobacco positions drove change. Information about damaging tobacco industry practices revealed through a series of American court cases gave ammunition to public health groups hoping to change public perceptions about tobacco.^9^^,^^12^

After the annulment of Directive 98/43/EC, legal activity increased, arguably becoming more adversarial than before. Between 2000 and 2004, 10 EU member states sued tobacco companies over smuggling in American courts, eventually resulting in a $1.25 billion dollar settlement in favor of the European states. The EU’s new TPD quickly faced a legal challenge from industry (C-491/01). But this time, the challenge failed. This happened, in part, because officials had gone further in the text of the Directive to frame the law as driven by market integration, highlighting the role of the directive in harmonizing disparate rules across member states. The Court determined that the TPD did indeed aim to facilitate market integration, and that having the protection of public health as a ‘decisive factor’ driving the law did not mean that the measure could not also support market integration. This thinking made its way into a subsequent string of jurisprudence and also influenced future actions taken by EU officials.

At this time, the EU and its member states also played a key role in facilitating the first international agreement on tobacco, the WHO Framework Convention on Tobacco Control (FCTC). The FCTC places binding (if not particularly enforceable) obligations on states to adopt more stringent tobacco control policies. Despite its lack of teeth, the agreement presents, in a legal form recognizable to courts, an international, evidence-based consensus in favor of a specific set of tobacco control policies. The European Community and all 28 EU member states are parties to the FCTC.

The involvement of the EU in facilitating the FCTC made a significant difference in the latest round of legal disputes that began in 2014. After five years of intense lobbying, corruption allegations and bargaining, the EU passed a revised version of the TPD in 2014 (TPD, 2014/40/EU). The new TPD augmented the powers of the EU on tobacco control in specific areas including the regulation of e-cigarettes, the prohibition of characterizing flavors including menthol, a requirement for larger mandatory warnings on packs. It also contained language emphasizing member states’ ability to go beyond the directive and implement plain packaging. The revised TPD thus demonstrates the extent to which the EU’s political institutions—particularly DG SANCO and the European Parliament—have developed political capacity in the area of public health since the early days of EU tobacco control policy. The text of the revised TPD is much less vulnerable than that of the original advertizing directive to challenges via the Court of Justice of the EU (CJEU), reflecting understanding on the part of the drafters of the difficult balance between of market harmonization and public health protections in EU law.^13^

The TPD was swiftly challenged in European courts by tobacco firms, involving domestic courts in the UK, Ireland and France as well as the CJEU (C-358/14, C-477/14, C547/14, C-151/17). The challenges focused on labeling measures in the context of certain member states’ decisions to implement plain packaging, provisions on e-cigarettes, the ban on characterizing flavors and the ban on sale of snus as well as questioning the purpose and appropriateness of the entire Directive.

In its responses, the CJEU ruled that the TPD is valid, supporting the EU’s authority to act in this area and rejecting claims that the measures were not appropriate to achieve the goals of the Directive. Significantly, the Court’s judgments refer extensively to the FCTC as a core part of the legal context. The Court uses FCTC obligations instrumentally to demonstrate an international political and scientific consensus in support of specific tobacco control policies, demonstrating just how important the FCTC is to global tobacco control efforts.

Further to supporting the EU’s ability to pass tobacco control regulations in general, the judgments thoroughly endorse the EU’s specific approach on some tricky issues. On e-cigarettes, an issue that countries around the world are addressing in very different ways, the Court found that a two-tier regulatory framework with different rules for cigarettes and e-cigarettes was appropriate given the different levels of risk that the two categories of products represent. On labeling, the Court affirmed the EU’s decision to allow member states to go beyond the TPD and introduce plain packaging. On characterizing flavors, the Court did not see a disconnect between the goal of market integration and that of protecting human health. Rather, the judgment affirms that the directive both achieves the goal of bringing different national rules into line with one another *and* is an appropriate measure for ensuring a high level of protection of human health.

But public health officials and advocates should not grow complacent in the face of these recent victories. Friction between the goals of market integration and public health protection still exists. In October 2014, an Italian law introducing minimum pricing for cigarettes was successfully challenged via the EU’s court system, with the CJEU determining that the law violated an EU Directive that sets an EU-wide tobacco excise duty (C-428/13, for a full account^13^) At the time of writing, a fourth challenge to the TPD questioning the EU’s ban on the sale of snus in all EU member states except Sweden, was still pending.

## Conclusion

Legalism is a form of governance that relies heavily on the authority of law to settle political disputes, with consequences for the ways in which governments make and advocates push for public health policies. Despite a series of losses for the industry, it looks likely that the EU and its member state governments will continue to be the targets of legal challenges over their tobacco control policies. In contrast, it looks less likely that government challenges against industry practices will emerge or succeed without a great deal of engagement from public health stakeholders and entrepreneurial government officials.

As long as the EU continues to pass tobacco control laws, litigation is likely to continue. This is not in itself a bad thing, given the extent to which the EU’s legal and political systems have evolved to support public health protections. But legalism does present a different set of challenges for health policymakers and public health advocates, and meeting these challenges will require significant resources, organizational capacity, legal expertise and strategy.

Public health groups around the world are attempting to harness legalism to their own ends, envisioning a wave of public health-led litigation designed to hold the industry criminally liable for its damaging practices. But in the first of these cases in Europe, the Netherlands Public Prosecution Service declined to proceed with charges against the industry, arguing that individuals have the ‘freedom of choice’ to smoke, and that consumers expose themselves to harmful tobacco products despite knowing the risks to their health.^14^

This misrepresentation of addiction as personal choice is all too familiar to American tobacco control activists. Similar misleading arguments were used in decades of expensive and bitter litigation in the USA to protect tobacco companies from liability charges brought by smokers and their families. Between the 1950s and 1990s, all such attempts to sue tobacco firms failed. Although litigation against the industry was ultimately successful, with legal costs to the industry driving up cigarette prices, the disclosure of important industry documents and valuable settlements in favor of state governments, we often forget that this success was preceded by years of expensive failure that was devastating to claimants and their families.

Other countries, most notably Canada, have tried to replicate the success of US public health advocates in turning adversarial legalism to their benefit. But although the European Commission has investigated the possibility of suing tobacco firms to recover health costs, differences between legal systems in the US and Europe may prove significant barriers to litigation.^15^

Without understanding legalism, its variants and how it works, it is not possible to understand how public health policy is made and sustained in contemporary Europe. Without the distinctive legalism of the ECJ, EU tobacco control law would have happened earlier and would not be tailored to internal market treaty bases. Without the rise of global and European legalism, the UK might have persisted with its informal approach, built around informal negotiations and trust with the tobacco industry. Public health policies legislated in contemporary Europe can be challenged under domestic law, European law and through various international legal frameworks, which means that understanding legalism is crucial for making policy.^16^ Further, if the trajectory of legalism creates procedural problems and distortions in the content of public health policy, then the content of law and its making is a public health problem. On the bright side, litigation is a public health tool as well, and an enforceable, justiciable right to health is a powerful political and legal weapon in its own right.

## Funding

This open access Supplement received funding under an operating grant from the European Union’s Health Programme (2014–2020).

*Conflicts of interest*: None declared.

**Key points**

- Legalism is a form of governance that relies on the authority of law to settle disputes.
- Tobacco control measures are heavily influenced by legalism.
- While legalism can support public health, it can also create procedural problems and distortions that derail public health policy.

## References

[1] Burke TF, Barnes J, editors. Varieties of Legal Order: The Politics of Adversarial and Bureaucratic Legalism. New York: Routledge, 2018.

[2] Kagan RA Adversarial Legalism: The American Way of Law. Cambridge, MA: Harvard University Press, 2003.

[3] Moran M The British Regulatory State: High Modernism and Hyper-Innovation. Oxford: OUP, 2004.

[4] Vogel S Freer Markets, More Rules: Regulatory Reform in Advanced Industrialized Countries. Ithaca: Cornell University Press, 1996.

[5] Kelemen D Eurolegalism. Cambridge, MA: Harvard University Press, 2011.

[6] Kelemen D Eurolegalism and democracy. J Common Mark Stud 2012;50:55–71.

[7] Greer SL, Jarman H European citizenship rights and European fiscal policies after the crisis. Gov Oppos 2018;53:76–103.

[8] McKee M, Hervey T, Gilmore A Public health policies In: Mossialos E, Permanand G, Baeten R, Hervey T, editors. Health Systems Governance in Europe. New York: Cambridge University Press, 2010: 231–81.

[9] Duina F, Kurzer P Smoke in your eyes: the struggle over tobacco control in the European Union. J Eur Public Policy 2004;11:57–77.

[10] Boessen S, Maarse H The impact of the treaty basis on health policy legislation in the European Union: a case study on the tobacco advertising directive. Health Serv Res 2008;8:77. 10.1186/1472-6963-8-77 PMC2358888 18397520

[11] Greer SL, Fahy N, Elliott HA, et al Everything You Always Wanted to Know about European Union Health Policy but Were Afraid to Ask. Brussels: WHO/European Observatory on Health Systems and Policies, 2014. 31855337

[12] Studlar D Tobacco control: the end of Europe’s love affair with smoking? In: Greer SL, Kurzer P, editors. European Union Public Health Policy: Regional and Global Perspectives. Abingdon: Routledge, 2012: 181–93.

[13] Hervey TK, McHale JV European Union Health Law. Cambridge: Cambridge University Press, 2015.

[14] Openbaar Ministerie. Analyse van de Tabaksaangifte, 2017 Available at: https://www.om.nl/vaste-onderdelen/zoeken/@102218/verder-onderzoek/Accessed (12 January 2018, date last accessed).

[15] GHK. A study on liability and the health costs of smoking: final report. DG SANCO, 2012. 2008/C6/046.

[16] Jarman H The Politics of Trade and Tobacco Control. Basingstoke: Palgrave Macmillan, 2015.

